# Spatio-Temporal Expression Pattern of Ki-67, pRB, MMP-9 and Bax in Human Secondary Palate Development

**DOI:** 10.3390/life11020164

**Published:** 2021-02-20

**Authors:** Tanja Šimić Bilandžija, Katarina Vukojević, Anka Ćorić, Ivna Vuković Kekez, Ivana Medvedec Mikić, Lidija Lasić Arapović, Natalija Filipović, Jasminka Anđelić, Mirna Saraga-Babić, Danijela Kalibović Govorko

**Affiliations:** 1 Department of Maxillofacial Surgery, University Hospital Center Mostar, 88000 Mostar, Bosnia and Herzegovina; tanjasb@gmail.com; 2 Department of Anatomy, Histology and Embryology, University of Split School of Medicine, 21000 Split, Croatia; katarina.vukojevic@mefst.hr (K.V.); natalija.filipovic@mefst.hr (N.F.); msb@mefst.hr (M.S.-B.); 3 Department of Medical Genetics, School of Medicine, University of Mostar, 88000 Mostar, Bosnia and Herzegovina; 4 Health Care Center Mostar, 88000 Mostar, Bosnia and Herzegovina; coricanka1@gmail.com (A.Ć.); lidija.lasic.a@gmail.com (L.L.A.); 5 Department of Orthodontics, University of Split School of Medicine, 21000 Split, Croatia; ikekez@mefst.hr; 6 Department of Endodontics and Restorative Dentistry, University of Split School of Medicine, 21000 Split, Croatia; imedvede@mefst.hr; 7 Department of Orthodontics, University of Montenergo, 81000 Podgorica, Montenegro; jasminka@ac.me

**Keywords:** palatogenesis, human, development, Bax, Ki-67, pRb, MMP-9

## Abstract

We analyzed the immunohistochemical expression of Ki-67, pRb, Bax, and MMP-9 during the human secondary palate formation (7th to 12th developmental weeks (DWs). The most significant proliferation was observed in the seventh DW with 32% of Ki-67-positive cells in the epithelium, while loose ectomesenchyme condensations (lec) and loose non-condensing ectomesenchyme (lnc) had only 18 and 11%, respectively (Kruskal–Wallis, *p* < 0.001), and diminished afterwards. Contrarily, pRb-positive cells were mostly located in the lnc (67%), with significant difference in comparison to epithelium and lec in all investigated periods (Kruskal–Wallis, *p* < 0.001). Ki-67- and pRb-positive cells co-expressed occasionally in all investigated periods. MMP-9 displayed a strong expression pattern with the highest number of positive cells during the seventh DW in the epithelium, with significant difference in comparison to lec and lnc (Kruskal–Wallis, *p* < 0.0001). The ninth DW is particularly important for the Bax expression, especially in the epithelium (84%), in comparison to lec (58%) and lnc (47%) (Kruskal–Wallis, *p* < 0.001). The co-expression of Bax and MMP-9 was seen only in the epithelium during seventh and ninth DWs. Our study indicates the parallel persistence of proliferation (Ki-67, pRb) and remodeling (MMP-9) that enables growth and apoptotic activity (Bax) that enable the removal of the epithelial cells at the fusion point during secondary palate formation.

## 1. Introduction

Palatogenesis, a highly ordered and regulated process of palate development, is initiated in humans in the sixth developmental week and is completed with palatal fusion by the 12th week of gestation [1,2,3]. During the process of neurulation, the cephalic neural crest cells (NCCs) migrate towards the future palatal region to populate, and together with the mesodermal cells, establish the five facial prominences that surround the primitive mouth: rostrally frontonasal prominence, caudally a pair of mandibular prominences, and a pair of maxillary prominences laterally [4,5]. NCCs are multipotent migratory cells that originate from the neuroectoderm at the lateral edges of the neural plate and eventually differentiate into fibrous, cartilage, and bony structures of the face and neck [6]. During the palatal morphogenesis, the frontonasal prominence divides to form lateral and medial nasal processes. The latter subsequently fuse with the maxillary prominences and form the primary palate. The posteriorly situated secondary palate arises as the paired palatal shelf outgrowths elevate and fuse in the midline, thus forming the medial epithelial seam (MES) in the midline that subsequently disintegrates [7,8,9]. The process of palatogenesis is highly complex; it involves an extensive network of signaling molecules, transcription factors, processes of proliferation and apoptosis, and crosstalk between the cells and matrix [10] that, if disrupted, leads to cleft palate formation, one of the most frequent human malformations [3,8]. The importance of the synchronized spatio-temporal process of palatal development can be observed by visualizing specific cell markers that appear during cell proliferation, differentiation or apoptosis, a highly important processes appearing in normal palatogenesis [8,11].

Among them, Ki-67 is a nuclear antigen that is used as a marker of proliferation, and as such, has been used in normal fetal as well as neoplastic tissues [4,12,13,14,15]. There is scarce evidence in the scientific literature about the role of Ki-67 protein in the development of the human palate [1,16], and the existing data suggest that proliferation decreases by the end of the fusion process. However, the exception of that process is observed in the non-condensed ectomesenchyme, where proliferation is increased due to the diversity of morphogenetic and differentiation processes important for the configuration of different tissues [1]. Defects in this process of development can lead to orofacial malformations [17].

The retinoblastoma protein (Rb) is controlled by a tumor suppressor gene and belongs to the retinoblastoma family of proteins with many phosphorylation and binding sites [18]. Over the G1 restriction point, Rb blocks the S phase of the cell cycle and cell growth [19], while the phosphorylated form (pRb) facilitates cell cycle progression [15,20]. The Rb protein also plays a central role in differentiation processes in various organs including the eyes, lenses, brain, peripheral nervous system, epidermis, melanocytes, hair cells, muscle, and liver cells [21]. The presence of pRB in the fetal murine tissue palatogenesis is scarcely investigated [22]. Investigation on experimental mice shows elevated pRb levels during the GD13-GD14 gestation periods, the time when the palatal shelves are re-orientating and merging [22].

Programed cell death or apoptosis is another vital process in palate development [4]. Its activation is dependent on different apoptotic pathways and is characterized by condensation of chromatin and cytoplasm, the appearance of apoptotic bodies and cell fragmentation [4,23]. Proapoptotic factor Bax is one of the products of the bcl-2 gene family, along with proapoptotic bac and anti-apoptotic bcl-2 and bcl-x proteins that are all involved in a wide variety of cellular activities [24]. A few studies have investigated cell proliferation and apoptosis in secondary palate formation on human embryos, while the majority of research has been performed on animal models or tissue cultures [1,4]. Bax involvement in human palatogenesis has not been investigated so far.

MMP-9 is an enzyme of the zinc metalloproteinase family (MMPs). It participates in the degradation of the extracellular matrix (ECM) in physiological processes of embryonic development, reproduction, angiogenesis, and bone development, as well as in wound healing, pathological processes, and metastatic dissemination [25,26,27,28]. The maintenance and remodeling of ECM is mediated by MMPs and its inhibitors [10]. Investigations into the role of MMP-9 in human embryonic palate development is not present in the world’s scientific literature, while analysis of the development of the secondary palate in animals indicates that MMP-9 increases at later stages of the palate development and decreases in expression following the end of palate fusion [29].

The formation of the human secondary palate requires a synchronized network of developmental events such as growth, remodeling, apoptosis, and fusion. Therefore, the aim of this study was to contribute more to the knowledge about the temporo-spatial distribution of proliferation (Ki-67, pRb) and remodeling (MMP-9) processes, and apoptotic activity (Bax) and their potential role in human secondary palate formation. Disturbance to these processes during the late embryonic and early fetal period might lead to cleft palate disorders.

## 2. Materials and Methods

### 2.1. Tissue Processing and Immunofluorescence Staining

Human conceptuses between the 7th and 12th developmental weeks were collected after ectopic pregnancies or spontaneous abortions from the Department of Pathology, University Hospital Center Split (Table 1).

All of the samples were examined for possible abnormalities and only samples without any abnormalities were included in the study. The study was conducted with approval of the Ethics Committee of the University Hospital Center Split, in accordance with the Helsinki Declaration [30]. Human head samples were dissected following fixation in 4% paraformaldehyde in phosphate buffer saline (PBS) and dehydration in graded ethanol. Paraffin-embedded tissue samples were serially sectioned (5 µm) and mounted on glass slides [23,31]. Immunohistochemistry was performed as we described previously [32,33]. Briefly, the sections were heated in citrate buffer (pH 6.0) in a microwave oven (13 min), cooled at room temperature and rinsed with PBS. Slides were incubated with protein block for 30 min following overnight incubation at 4 °C with an appropriate primary antibody mixture—mouse monoclonal anti-human-MMP-9 (MA5-14228), rabbit monoclonal anti-human pRb (ab173289, Abcam, UK), rabbit polyclonal anti-human Ki-67 antigen (AB9260, Chemicon, Temecula, CA, USA), and anti-mouse Bax antigen (AB 2915, Chemicon, Temecula, CA, USA). After incubation with primary antibodies, slides were rinsed with PBS and incubated in an appropriate combination of secondary antibodies—anti-mouse Alexa Fluor 594 (RED; ab150108, Abcam, UK), anti-rabbit Alexa Fluor 488 (GREEN; ab150073, Abcam, UK), and anti-mouse Alexa Fluor 488 (GREEN; ab150105, Abcam, UK). For the nuclei staining, we used 4′,6-diamidino-2-phenylindole (DAPI). Images of tissue sections were captured by an Olympus (Tokyo, Japan) BX51 microscope equipped with a Nikon DS-Ri1 camera (Nikon Corporation, Tokyo, Japan) and assembled with Adobe Photoshop (Adobe Systems, MI, USA).

### 2.2. Quantitative and Semi-Quantitative Analysis

A total of 10 non-overlapping fields were taken using 40× objective magnification. The cell count was performed using ImageJ software (National Institutes of Health, Bethesda, MD, USA). In each microphotograph, the number of Ki-67-, pRb-, MMP-9-, and Bax-positive cells per 100 cells in total was quantified to obtain the percentage of immunoreactivity for each marker per specific palate structure (epithelium, loose ectomesenchyme condensations and loose non-condensing ectomesenchyme). Those percentages of immunoreactivity per specific structure were then compared between all the samples, as we described previously [34,35,36]. One-way ANOVA and Turkey’s post hoc test (GraphPad Software, San Diego, CA, USA) were used for statistical analysis to examine the difference between epithelium, loose ectomesenchyme condensations, and loose non-condensing ectomesenchyme in the 7th, 9th, and 12th week of development. The level of statistical significance was set at *p* < 0.05.

The antibody staining intensity was evaluated by six independent investigators. A score of 3 indicated strong staining intensity, 2 indicated moderate staining intensity, 1 indicated mild staining intensity, and 0 indicated no staining.

## 3. Results

### 3.1. Phases of Normal Secondary Palate Development

**Figure 1:**
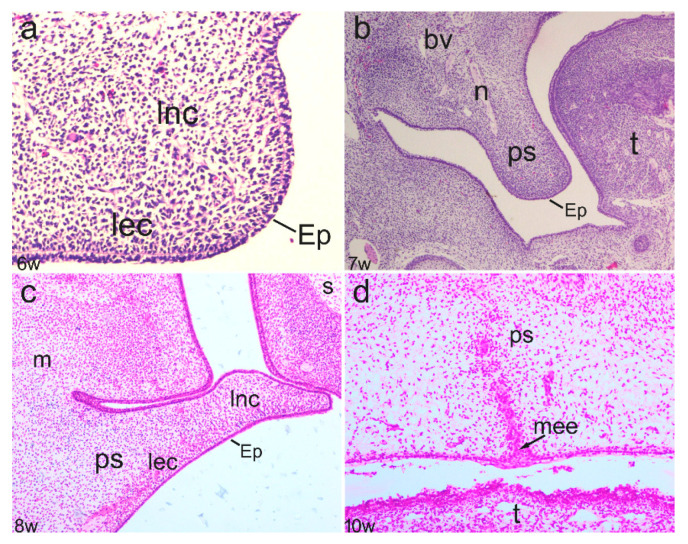
Frontal section through different developmental stages of secondary palate in the 6th week (**a**), 7th week (**b**), 8th week (**c**) and 10th week (**d**) of human conceptuses—epithelium (Ep), loose ectomesenchyme condensations (lec), loose non-condensing ectomesenchyme (lnc), medial edge epithelium (mee), palatal shelves (ps), nerves (n), blood vessels (bv), tongue (t), maxilla (m), nasal septum (s).

In the early stages of secondary palate development (sixth week), the initial protrusion of palatal shelves consisting of centrally positioned ectomesenchyme and surface oral epithelium was observed. However, palatal shelves were widely separated, without signs of palatal structure differentiation (Figure 1a). In the seventh developmental week, further growth of the palatal shelves continued, but shelves were oriented downwards on either side of the tongue (Figure 1b). The entrance of nerves and blood vessels was observed in the ectomesenchyme, which, in certain places, showed signs of condensation. During further development, the palatal shelves elevated and became oriented perpendicularly to the maxillary processes. The initial signs of intramembranous ossification were observed in the developing maxillae in the eighth week of development (Figure 1c). Finally, the palatal shelves fused to form the secondary palate. At the fusion points, remnants of epithelium covering the palatal shelves were still present in the 10th developmental week (Figure 1d).

### 3.2. Expression Patterns of Ki-67, pRb, Bax, and MMP-9 in the Embryonic and Early Fetal Development of the Human Secondary Palate Formation (Figure S1)

#### 3.2.1. The Sixth to Seventh Week of Development

**Figure 2:**
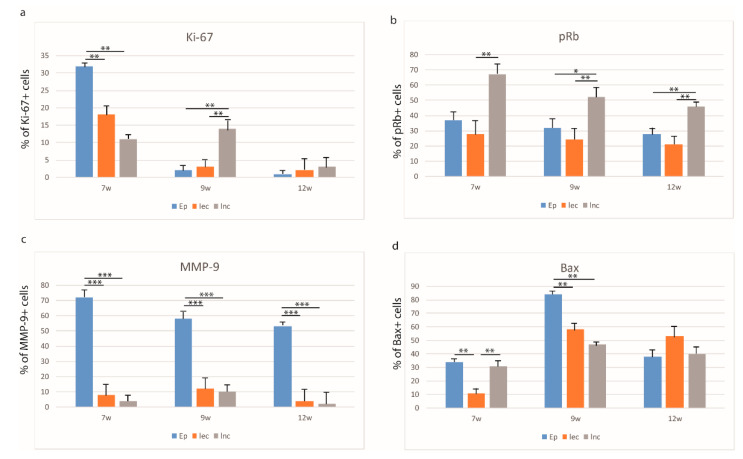
Distribution of Ki-67 (**a**) pRb- (**b**), MMP-9- (**c**) and Bax- (**d**) positive cells in the epithelium (Ep), loose ectomesenchyme condensations (lec), loose non-condensing ectomesenchyme (lnc) in formation of the secondary palate (7th–12th week). Data are shown as mean ± SD. Significant differences (Kruskal–Wallis) indicated by * *p* < 0.05, ** *p* < 0.001, *** *p* < 0.0001.

In the seventh week of human development, palatal shelves that were protruding from the maxillary prominences started to approach each other in the midline, thus contributing to the secondary palate development and morphogenesis. Ki-67-positive cells were mostly located in the surface epithelium of the palatal shelves (32%), while loose ectomesenchyme condensations and loose non-condensing ectomesenchyme contained only 18 and 11% of Ki-67-positive cells (Kruskal–Wallis, *p* < 0.001) (Figure 2a). In contrast, pRb-positive cells were mostly located in the loose non-condensing ectomesenchyme (67%), while the epithelium and loose ectomesenchyme condensations had 37 and 28%, respectively (Kruskal–Wallis, *p* < 0.001) (Figure 2b). Ki-67- and pRb-positive cells in all structures had a strong staining intensity (Table 2.)

**Figure 3:**
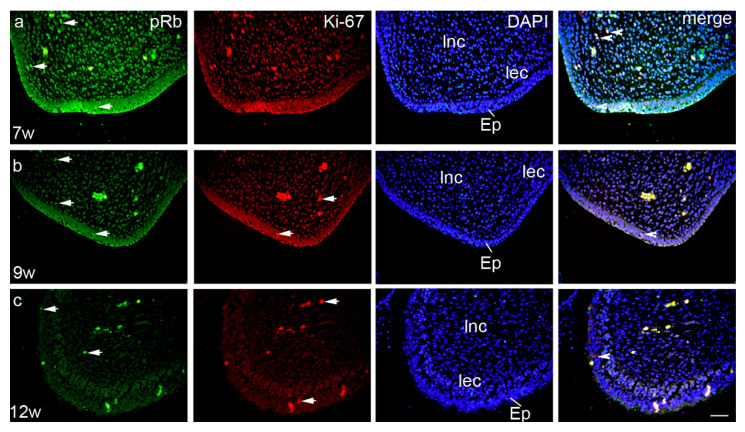
Transversal section through the secondary palate of 7 weeks (**a**), 9 weeks (**b**) and 12 weeks (**c**) old human embryo: pRb- (green) and Ki-67- (red) positive cells (arrows) can be seen in the epithelium (Ep), loose non-condensing ectomesenchyme (lnc), and loose ectomesenchymal condensations (lec). Some cells are co-expressing both markers (arrowheads). Blue nuclear staining (DAPI). Scale bar 25 µm.

**Figure 4:**
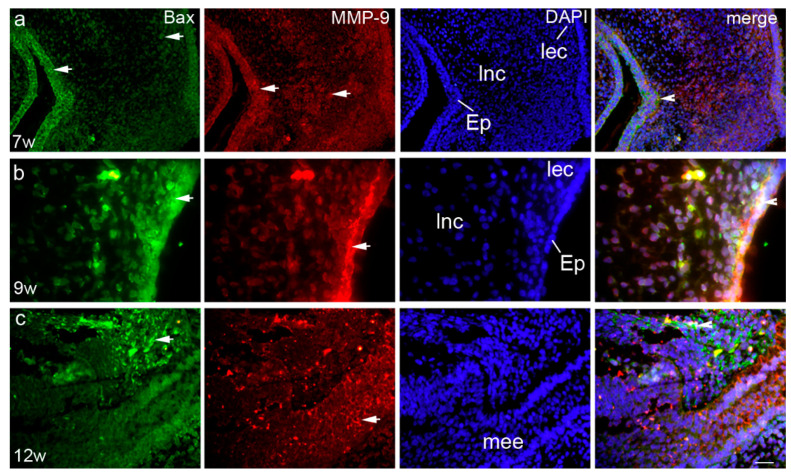
Transversal section through the secondary palate of 7 weeks (**a**), 9 weeks (**b**) and 12 weeks (**c**) old human embryo: Bax- (green) and MMP-9-(red) positive cells (arrows) can be seen in the epithelium (Ep), loose non-condensing ectomesenchyme (lnc), and loose ectomesenchymal condensations (lec), medial edge epithelium (mee). Only a few cells in the basal part of epithelium are co-expressing both markers (arrowheads). Blue nuclear staining (DAPI). Scale bar 25 µm (**a**) and (**c**), 10 µm (**b**).

Ki-67- and pRb-positive cells co-expressed occasionally in all investigated areas—2% in epithelium, 5% in loose non-condensing ectomesenchyme, and 1% in loose ectomesenchyme condensations (Figure 3a). At that developmental stage, MMP-9 displayed a strong expression pattern and moderate staining intensity, with the highest number of positive cells in the epithelium (72%), while loose ectomesenchyme condensations and loose non-condensing ectomesenchyme had only 8 and 4% of positive cells and mild staining intensity, respectively (Kruskal–Wallis, *p* < 0.0001) (Figure 2c, Table 2). Bax-positive cells were observed in the epithelium and the loose non-condensing ectomesenchyme (34 and 31% of positive cells, respectively), while in the loose ectomesenchyme condensations, there were only 11% of positive cells (Kruskal–Wallis, *p* < 0.001) (Figure 2d). Epithelial cells showed strong staining intensity, while loose non-condensing ectomesenchyme and loose ectomesenchyme condensations had mild staining intensities (Table 2). Co-expression of Bax and MMP-9 was seen only in the epithelium in 8% of cells (Figure 4a).

#### 3.2.2. The Eighth to Ninth Week of Development

In the early fetal period, palatal shelves attained a horizontal position. Ki-67-positive cells with strong staining intensity were mostly located in the loose non-condensing ectomesenchyme (14%), while in the epithelium and loose ectomesenchyme condensations, Ki-67-positive cells were seen only occasionally (2 and 3%, respectively) (Kruskal–Wallis, *p* < 0.001) (Figure 2a, Table 2). In contrast, the number of pRb-positive cells remained highest in the loose non-condensing ectomesenchyme, but started to decline to 52%, and there was a significant difference in comparison to the loose ectomesenchyme condensations (24%) (Kruskal–Wallis, *p* < 0.001). A similar difference was observed for the epithelium and loose non-condensing ectomesenchyme (32%) (Kruskal–Wallis, *p* < 0.05) (Figure 2b). pRb-positive cells had moderate staining intensity in all structures (Table 2). Ki-67- and pRb-positive cells co-expressed only occasionally in 2% of epithelial cells (Figure 2b). MMP-9 continued to display a strong expression pattern in the epithelium. Thus, the loose ectomesenchyme condensations and loose non-condensing ectomesenchyme had only 12 and 10% of positive cells, respectively, in comparison to the epithelium (58%) (Kruskal–Wallis, *p* < 0.0001) (Figure 2c). The ninth week of development was particularly important for the Bax expression, especially in the epithelium (84%, strong staining intensity), but also with the highest rate in both the loose ectomesenchyme condensations (58%, moderate staining intensity) and loose non-condensing ectomesenchyme (47%, mild staining intensity) (Kruskal–Wallis, *p* < 0.001) (Figure 1d, Table 2). As in the seventh week of development, the co-expression of Bax and MMP-9 was seen only in the epithelium in 12% of cells (Figure 4b).

#### 3.2.3. The 12th Week of Development

In the 12th week of development, palatal shelves completely fused in the midline and the proliferation rate diminished accordingly. Namely, Ki-67-positive cells were on the minimal level in all three analyzed areas, but with strong staining intensity (Figure 2a, Table 2). However, pRb-positive cells displayed the same pattern as in previous developmental periods, but with a lower number of positive cells. Differences between loose ectomesenchyme condensations and loose non-condensing ectomesenchyme and between the epithelium and loose non-condensed ectomesenchyme have been observed (Kruskal–Wallis, *p* < 0.001) (Figure 2b). Ki-67- and pRb-positive cells co-expressed occasionally in all investigated areas—2% in the epithelium and as low as 1% in both the loose non-condensing ectomesenchyme and the loose ectomesenchyme condensations (Figure 3c). The number of MMP-9-positive cells declined in this period, but still with 53% (strong staining intensity) of positive cells in the epithelium, and 4 and 2% (moderate staining intensity both) of positive cells in the loose ectomesenchyme condensations and loose non-condensing ectomesenchyme, respectively (Kruskal–Wallis, *p* < 0.0001) (Figure 2c, Table 2). The Bax expression started to decline in the 12th week of development, with the highest number of Bax-positive, strong-intensity staining cells in the loose ectomesenchyme condensations (53%) in comparison to the other two areas (Figure 2d, Table 2). The co-expression of Bax and MMP-9 was not seen in any investigated area in this developmental period (Figure 4c).

## 4. Discussion

The secondary palate formation occurs between the 6th and 12th developmental week and the main prerequisite for the fusion of all prominences involved is their proper spatial orientation and shedding of the surface epithelium at the contact fusion point (the nasal fin and/or the median seam epithelium) [37]. Accordingly, the number of Ki-67- and pRb-positive cells in our study decreased during development, with the lowest proliferation index in the epithelium during the 12th week of development (Supplementary Figure S1). Those results indicate that after palatal shelves have fused, proliferation diminished in order to be replaced by processes of apoptosis in the surface epithelium. Additionally, a decreased pRb expression in the epithelium during the same period might suggest its association with the initial differentiation of the oral and nasal epithelium cells into typical ciliated columnar or stratified squamous epithelium. At later developmental stages, the persistence of a higher expression of pRb in the non-condensing ectomesenchyme might imply its participation in intramembranous bone formation, which is, at the beginning, associated with increased cell proliferation of future osteoblasts. Therefore, the fine balance between the cell proliferation and pRb protein, which is supposed to facilitate the cell cycle, might guide the proper differentiation of different tissues in the ectomesenchyme, including bone, connective tissue, and muscles. The synchronized acting of the proliferation and pRb expression is important in the prevention of cleft palate development. Namely, it was suggested that retinoic acid induce anti-proliferative activity by inhibition of Rb phosphorylation in mouse embryonic palatal mesenchymal cells, which might account for the pathogenesis of a cleft palate [38,39]. Upon palatal shelf fusion, the medial epithelial seam (MES) needs to disappear in order to enable further fusion of the ectomesenchymal parts of the palatal processes (Supplementary Figure S1). Recent studies on cell cultures and experimental animals showed that programmed cell death (apoptosis) and epithelial–mesenchymal transformation (EMT) may both be required to complete MES disintegration [40,41]. Previously, we reported on the fusion of the secondary human palate and showed the importance of simultaneous proliferative and apoptotic activity during MES disappearance [1]. Other investigations on the role of apoptotic and anti-apoptotic factors during the human jaw development have further stressed the importance of the balance between those factors for the proper differentiation of jaw cartilage and bone [4,23]. In our present study, proliferating and Bax-positive apoptotic cells were found both in the epithelia and ectomesenchymal part of the developing palate, showing differences in intensity and distribution that could be associated with the disintegration of the surface epithelium and differentiation and morphogenesis of different structures in the palate ectomesenchymal core. Therefore, we propose that the condensing ectomesenchyme might be associated with the differentiation of the connective tissue component of palate mucosa (*lamina propria*), while the non-condensing ectomesenchyme probably gives rise to the cartilaginous and bony components of the palate. Cell death is mainly involved in MES disintegration during normal palatogenesis; the death of some medial epithelial edge (MEE) cells contributes to palatal mesenchyme continuity, but apoptotic cells are hardly found in the palate mesenchyme except those in close proximity to the MES [9]. Before adhesion, the MEE comprises two layers of epithelium—a flat periderm and a cuboidal basal MEE. After palatal shelf reorientation, the periderm cells start to change morphologically; they swell and show features of cell death which then detach from the surface. The basal MEE cells become exposed and ready to make contact with the MEE of the opposing palatal shelf [42]. Contact of the opposing MEE commences in the region of the second ruga (middle third of the palate) and extends to both the anterior and posterior ends of the palate. In the anterior part, the majority of periderm cells are shed by the time of contact, although some are trapped between the opposing basal MEE. Accordingly, the MES is initially a two-layer-thick structure which becomes thinner to reach a single layer by intercalation of the MES cells from both sides of the shelf. In our study, at the ninth week of development, expression of the pro-apoptotic Bax protein was particularly high, thus suggesting an initial shift toward apoptosis, which is required to remove the MES at the palatal junction. Decrease in the Bax levels at 12th week of development might suggest that executive pro-apoptotic markers, such as caspase-3, assume a leading role in the degradation of the MES. The co-expression of Bax and MMP-9 in the epithelium at the seventh week of development may indicate their joint role in apoptosis. Namely, Chen et al. investigated diabetic retinopathy and found out that MMP-9 may induce cell apoptosis via regulating Ang2 or targeting apoptotic proteins, such as Bax2, Bcl2, cleaved PARP and cleaved caspase-3 in rat retina cell culture [43]. In addition, the application of an MMP-9 blocking antibody or the genetic deletion of MMP-9 inhibits the axonal outgrowth, migration, and apoptosis of granule cell precursors in the developing cerebellum [44]. The extracellular matrix molecules seem to play a key role in the morphogenesis of the palate. It is believed that MMPs 2 and 9 in samples in vivo through epithelial mesenchymal interactions mediate intensive extracellular degradation [45]. The overall high levels of MMP-9 in our study might contribute to the reorganization of palatal shelves, as well as the transformation of epithelial cells from the MES into the mesenchymal cells during the palate fusion. This finding is in line with the study of Morris-Wiman et al. on murine secondary palate morphogenesis [46]. This human palate study suggests that MMP-9 and its inhibitors are the main regulatory proteins of extracellular matrix (ECM) expression in unilateral cleft lip and/or palate development [10]. The morphogenesis of a cleft lip and/or palate is closely followed by changes in the extracellular matrix that promote cell migrations, differentiation, cells interaction, and tissue resorption. It seems that the imbalance of the matrix metalloproteinases can lead to a cleft palate since their main role is the remodeling of the extracellular matrix and basal cell membrane modulation of the MES [10,47]. In conclusion, the proliferation of palatal shelves and their fusion together with the disappearance of MES promoted by apoptosis and remodeling are crucial steps of palatogenesis. Complete MES disintegration is the final phase of palatal integrity with surrounding mesenchymal cells. Improper crosstalk between proliferative, apoptotic, and remodeling factors used in this study may lead to disruption of palate shelf fusion and subsequent palatal cleft formation.

## Figures and Tables

**Table 1:** The age and number of the human embryos and fetuses analyzed in this study.

Age (weeks)	CRL (mm)	Carnegie Stage	Biparietal Diameter (mm)	No. of Conceptuses
6	13–14	16–17	/	5
7	15	18	/	5
8	18–19	21–23	/	5
9	20	/	/	5
10	/	/	18–21	5
12	/	/	26–28	5

**Table 2:** Staining intensity to specific antibodies in formation of the secondary palate during the 7th, 9th, and 12th weeks of development.

Structure	Antibodies											
	pRb			Ki-67			MMP-9			Bax		
	7w	9w	12w	7w	9w	12w	7w	9w	12w	7w	9w	12w
Ep	3	2	1	3	3	3	2	3	3	3	3	1
lec	3	2	2	3	3	3	1	1	2	1	2	3
lnc	3	2	2	3	3	3	1	1	2	1	1	3

3: strong staining intensity; 2: moderate staining intensity; 1: mild staining intensity; 0: no staining, epithelium (Ep), loose ectomesenchyme condensations (lec), loose non-condensing ectomesenchyme (lnc); w: week of development.

## Data Availability

The data presented in this study are available on request from the corresponding author.

## Supplementary Materials

The following are available online at https://www.mdpi.com/2075-1729/11/2/164/s1, Figure S1: Schematic drawing of different developmental stages of secondary palate and percentage of Ki-67-, pRb-, Bax- and MMP-9-positive cells in the 7th week (**a**), 9th week (**b**) and 12th week (**c**) of human conceptuses.
