# Supplementary material for: Spatio-Temporal Expression Pattern of Ki-67, pRB, MMP-9 and Bax in Human Secondary Palate Development
Source: Life (Basel). 2021 Feb 20;11(2):164. doi: 10.3390/life11020164 (PMC7924200; doi:10.3390/life11020164)
Supplement: Supplementary file 1 [file life-11-00164-s001.pdf]

life

MDPI

*Article*

# Spatio-temporal Expression Pattern of Ki-67, pRB, MMP-9 and Bax in Human Secondary Palates Development

**Tanja Šimić Bilandžija [1,†], Katarina Vukojević [2,3,†], Anka Ćorić [4], Ivna Vuković Kekez [5], Ivana Medvedec Mikić [6], Lidija Lasić Arapović [4], Natalija Filipović [2], Jasminka Anđelić [7], Mirna Saraga-Babić [2] and Danijela Kalibović Govorko [5,*]**

**Supplementary Materials:**

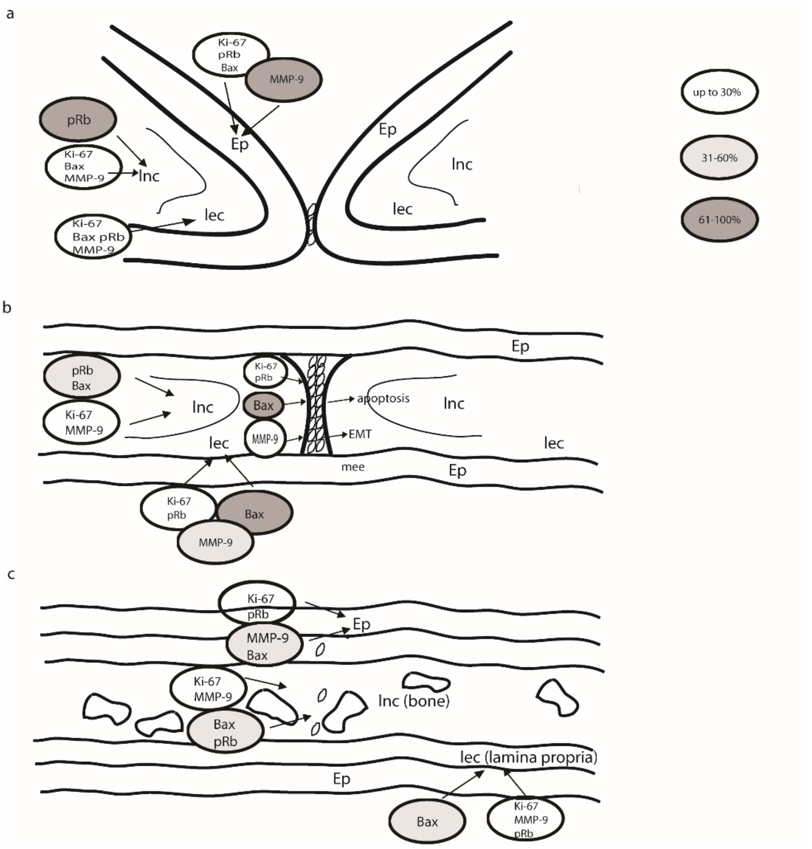


***Supplementary S1.*** Schematic drawing of different developmental stages of secondary palate and percentage of Ki-67, pRb, Bax and MMP-positive cells in the 7th week (**a**), 9the week (**b**) and 12th week (**c**) of human conceptuses: epithelium (Ep), loose ectomesenchyme condensations (lec), loose non-condensing ectomesenchyme (lnc), medial edge epithelium (mee).
